# *ASXL1* but Not *TET2 Mutations* Adversely Impact Overall Survival of Patients Suffering Systemic Mastocytosis with Associated Clonal Hematologic Non-Mast-Cell Diseases

**DOI:** 10.1371/journal.pone.0085362

**Published:** 2014-01-21

**Authors:** Gandhi Damaj, Magalie Joris, Olivia Chandesris, Katia Hanssens, Erinn Soucie, Danielle Canioni, Brigitte Kolb, Isabelle Durieu, Emanuel Gyan, Cristina Livideanu, Stephane Chèze, Momar Diouf, Reda Garidi, Sophie Georgin-Lavialle, Vahid Asnafi, Ludovic Lhermitte, Christian Lavigne, David Launay, Michel Arock, Olivier Lortholary, Patrice Dubreuil, Olivier Hermine

**Affiliations:** 1 Service d'Hématologie, Centre Hospitalier Universitaire, Hôpital Sud; Amiens, France; 2 Centre de Référence des Mastocytoses, Faculté de Médecine et AP-HP Necker-Enfants Malades, Paris, France; 3 CNRS UMR 8147 and Institut Imagine, AP-HP, Hôpital Necker-Enfants Malades, Paris, France; 4 Service d'Hématologie Adulte, Université Paris Descartes, Paris Sorbonne Cité, Faculté de Médecine et AP-HP Necker-Enfants Malades, Paris, France; 5 Inserm, U1068, CRCM, (Signaling, Hematopoiesis and Mechanism of Oncogenesis); Institut Paoli-Calmettes,Marseille; Aix-Marseille Univ; CNRS, UMR7258, Marseille, France; 6 Service d'Anatomo-pathologie, Université Paris Descartes, Paris Sorbonne Cité, Faculté de Médecine et AP-HP Necker-Enfants Malades, Paris, France; 7 Service d'Hématologie, Centre Hospitalier Universitaire, Reims, France; 8 Service de médecine interne, Groupe Hospitalier Sud. Hospices Civils, Lyon, France; 9 Service d'Hématologie et thérapie cellulaire, CIC INSERMU202, Centre Hospitalier Universitaire, Tours, France; 10 Département de Dermatologie, Centre Hospitalier Universitaire, Toulouse, France; 11 Service d'Hématologie, Centre Hospitalier Universitaire, Caen, France; 12 Département de bio-statistiques et de Recherche clinique, Centre Hospitalier Universitaire, Amiens, France; 13 Service d'Hématologie, Centre Hospitalier, St Quentin, France; 14 Service de Médecine Interne, Hôpital Tenon, Assistance Publique-Hôpitaux, Université Pierre et Marie Curie, Paris, France; 15 Laboratoire d'hématologie Biologique et UMR CNRS 8147, Université Paris Descartes, Paris Sorbonne Cité, Faculté de Médecine et Assistance Publique-Hôpitaux de Paris (AP-HP) Necker-Enfants Malades, Paris, France; 16 Service d'Hématologie, Centre Hospitalier Universitaire, Angers, France; 17 Service de Médecine Interne, CHRU, Lille, France; 18 CNRS UMR 8113, Laboratoire de Biologie et Pharmacologie Appliquée, Ecole Normale Supérieure, Cachan, France; 19 Laboratoire Central d'Hématologie, Groupe Hospitalier Pitié-Salpetrière, Paris, France; 20 Service de Médecine Interne et de Maladie Infectieuses, Université Paris Descartes, Paris Sorbonne Cité, Faculté de Médecine et AP-HP Necker-Enfants Malades, Paris, France; Institut national de la santé et de la recherche médicale (INSERM), France

## Abstract

Systemic mastocytosis with associated hematologic clonal non-mast cell disease (SM-AHNMD) is a rare and heterogeneous subtype of SM and few studies on this specific entity have been reported. Sixty two patients with Systemic mastocytosis with associated hematologic clonal non-mast cell disease (SM-AHNMD) were presented. Myeloid AHNMD was the most frequent (82%) cases. This subset of patients were older, had more cutaneous lesions, splenomegaly, liver enlargement, ascites; lower bone mineral density and hemoglobin levels and higher tryptase level than lymphoid AHNMD. Defects in *KIT*, *TET2*, *ASXL1* and *CBL* were positive in 87%, 27%, 14%, and 11% of cases respectively. The overall survival of patients with SM-AHNMD was 85.2 months. Within the myeloid group, SM-MPN fared better than SM-MDS or SM-AML (p = 0.044,). In univariate analysis, the presence of C-findings, the AHNMD subtypes (SM-MDS/CMML/AML *versus* SM-MPN/hypereosinophilia) (p = 0.044), Neutropenia (p = 0.015), high monocyte level (p = 0.015) and the presence of *ASXL1* mutation had detrimental effects on OS (p = 0.007). In multivariate analysis and penalized Cox model, only the presence of *ASXL1* mutation remained an independent prognostic factor that negatively affected OS (p = 0.035). SM-AHNMD is heterogeneous with variable prognosis according to the type of the AHNMD. ASXL1 is mutated in a subset of myeloid AHNMD and adversely impact on OS.

## Introduction

Mastocytosis is a heterogeneous group of disorders characterized by abnormal growth and accumulation of mast cells (MCs) in one or more organ systems. Indolent forms of mastocytosis are the most frequent followed by systemic mastocytosis (SM) with associated clonal hematologic non-MC lineage disease (SM-AHNMD) which constitutes a sub-category of aggressive SM [1]–[3].

Although most of these patients display *KIT* mutations, little is known about the clinical and prognostic relevance of other gene defects in SM-AHNMD. Only studies with a small number of SM cases, including few cases of SM-AHNMD, have been reported in 23 and 8 patients respectively [4], [5]. The overall prognosis for this disease is poor as compared to indolent SM patients. Furthermore, the subtype of the AHNMD has a prognostic value [6]. For example, patients with associated AML or MDS have a worse prognosis compared to patients with SM-MPN [6]. Nevertheless, some SM-AHNMD patients have a chronic course that seems to embrace the course of the associated hematologic disease.

The prognostic value of gene mutations has not been evaluated in large studies. In one study, reporting on 42 patients with SM, *TET2* mutations were not found to alter the prognosis of the disease [4], whereas two other studies showed that the presence of *TET2* mutations conferred a poor prognosis to patients with aggressive SM [5], [7].

In the present study, we report on 62 patients with SM-AHNMD with the following aims: 1/ To describe the clinical and laboratory characteristics of myeloid and lymphoid AHNMD patients; 2/ To evaluate the occurrence of genetic mutations such as *KIT* D816V and *JAK2* V617F as well as *TET-2*, *ASXL1, and CBL*; 3/ To evaluate a possible impact of these genetic mutations on the clinical and laboratory characteristics of SM-AHNMD patients as well as on their prognosis.

## Patients and Methods

This retrospective study was approved by the Institutional Review Board of Necker Enfants-Malades Hospital, and was carried out according to the Helsinki Declaration. Written Informed consent was obtained from adult patients or parents or next of kin for teenage patients at the first or second hematology clinic appointment. Patients aged 16 years or more, diagnosed with SM-AHNMD according to the WHO criteria [1] for mastocytosis and other hematologic neoplasms were eligible. Mutations analyses were centrally performed for all patients (KH, ES, PD). Mutations analyses were done on bone marrow samples and/or skin biopsy as previously described using a highly sensitive technique for *KIT* D816V [8], *JAK-2* [9] and *TET-2* [10]. *ASLX1* mutations status was detected in blood and/or marrow as already described [11].

Overall survival (OS) was defined as the time between diagnosis of AHNMD and the date of death or last follow-up. Survival distributions were estimated using the Kaplan-Meier method. Univariate and multivariate Cox proportional hazards model were used to assess prognostic factors of OS and to compute hazard ratios (HRs) and their 95% confidence interval (95% CI). Variables tested in the univariate model were those listed in table 1. Variables eligible for the multivariate Cox model were those having a p-value<0.15 in univariate Cox model because most of the variables significant at 10% other than ASXL1 were highly correlated with each other; thus limiting multicollinearity in the multivariate model. Proportional hazard assumption was tested using Schoenfeld residuals test. P values were two sided and values <0.05 were considered significant. Due to the low proportion of event in our study, a complementary analysis using a penalized ridge Cox model [12] was applied with a shrinkage parameter equal to 3. The SAS software version 9.2® (SAS Institute, Cary, NC) was used for all analysis.

**Table 1:** Demographic, clinical and biological characteristics of patients with myeloid and lymphoid SM-AHNMD patients at inclusion.

	Total n = 62 (%)	Myeloid AHNMD n = 51 (%)	Lymphoid AHNMD n = 11 (%)	*P*
Age (years); median (range)	64 (16–84)	67 (16–84)	53 (23–74)	*0.032*
Sex; n (%)				*1.0*
Female	23 (37)	19 (37)	4 (36)	
Male	39 (63)	32 (63)	7 (64)	
CM subtypes; n (%)				*0.038*
UP	23 (38)	16 (33)	7 (64)	
TEMP	9 (15)	8 (16)	1 (9)	
DCM	7 (12)	6 (12)	1 (9)	
No lesions	21 (35)	19 (39)	2 (18)	
1C-findings; n (%)				*0.045*
Yes	35 (56)	32 (63)	3 (27)	
No	27 (44)	19 (27)	8 (73)	
C-findings excluding cytopenia; n (%)				*1.0*
Yes	16 (26)	13 (25)	3 (27)	
No	46 (74)	38 (75)	8 (73)	
2Mast cell activating symptoms				*0.394*
Yes	51 (82)	43 (84)	8 (73)	
No	11 (18)	8 (12)	3 (27)	
Pruritus				*0.167*
Yes	23 (38)	17 (33)	6 (60)	
No	38 (62)	34 (67)	4 (40)	
Pollakiuria; n (%)				*0.142*
Yes	8 (13)	5 (9)	3 (27)	
No	54 (87)	46 (91)	8 (73)	
Neuropsychological symptoms; n (%)				*0.685*
Yes	13 (21)	10 (18)	3 (27)	
No	48 (79)	41 (72)	8 (73)	
Digestive symptoms; n (%)				*1.0*
Yes	30 (61)	26 (60)	4 (67)	
No	19 (39)	17 (40)	2 (33)	
Hepatomegaly; n (%)				*0.149*
Yes	27 (49)	25 (52)	2 (22)	
No	30 (51)	23 (48)	7 (78)	
Splenomegaly; n (%)				*0.003*
Yes	38 (68)	36 (76)	2 (22)	
No	18 (32)	11 (24)	7 (78)	
Ascites; n (%)				*0.046*
Yes	17 (30)	17 (35)	0	
No	40 (70)	31 (65)	9 (82)	
Lymph nodes enlargement; n (%)				*1.0*
Yes	21 (37)	18 (37)	3 (33)	
No	36 (63)	30 (63)	6 (67)	
BMD; n (%)				*0.045*
Normal	29 (52)	27 (56)	2 (25)	
Osteoporosis	14 (25)	9 (19)	5 (63)	
Osteopenia	13 (23)	12 (25)	1 (12)	
Hemoglobin, (mean; range, g/dl)	11.6 (7.7–16.9)	11.4 (7.7–16.9)	12.9 (11.1–13.5)	0.029
Neutrophils (mean; range,10^9^/l)	4.2 (0.1–24.3)	4.2 (0.1–24.3)	4.0 (1.5–8.08)	0.547
Eosinophils (mean; range,10^9^/l)	0.59 (0–6.4)	0.6 (0.0–6.4)	0.2 (0.0–2.4)	0.160
Basophils (mean; range,10^9^/l)	0.0 (0–0.5)	0.0 (0–0.5)	0.0 (0–0.05)	0.399
Monocytes (mean; range,10^9^/l)	0.7 (0.08–6.96)	1.2 (0.2–6.96)	0.5 (0.08–0.8)	0.028
Lymphocyte (mean; range,10^9^/l)	1.6 (0.1–5.20)	1.6 (0.1–5.20)	2.2 (1.1–2.9)	0.149
Platelets (mean; range,10^9^/l)	181 (10–1036)	133 (10–1036)	250 (170–466)	0.126
Tryptase (ng/ml)	137 (10–697)	169 (10–697)	50 (19.4–204)	0.046
LDH (mean; range,UI/l)	292 (126–737)	302 (141–737)	213 (126–329)	0.020
*KIT* genotype; n (%)				0.674
D816 positive	53 (85)	44 (86)	8 (75)	
Non D816V	1 (2)	1 (2)	0	
WT	8 (13)	6 (12)	2 (25)	
*TET2* mutations; n (%)				0.163
Yes	12 (27)	12 (32)	0	
No	32 (73)	25 (68)	7	
*ASXL1* mutations; n (%)				0.567
Yes	6 (14)	6 (17)	0	
No	37 (86)	30 (83)	7	
*JAK2* mutations; n (%)				0.567
Yes	3 (13)	3 (7.5)	0	
No	44 (87)	37 (92.5)	7	
*CBL* mutations; n (%)				0.778
Yes	3 (11.5)	3 (12.5)		
No	23 (88.5)	21 (87.5)	0	

1 C-findings according to WHO classification.

2 including fatigue, headache, flushes, fever, hypotension, choc, syncope, WHO; world health organization, CM; cutaneous mastocytosis, AHNMD; associated clonal hematologic non-mast cell lineage disease, UP; urticaria pigmentosa, TEMP; telengietasia eruptive macularis persistans, DCM; diffuse cutaneous mastocytosis, BMD; bone mineral density.

## Results and Discussion

Sixty-two SM-AHNMD patients were analyzed. Myeloid AHNMD were the most frequent type of AHNMD encountered (82% of the patients) (table S1). MDS was the leading myeloid disease (29%) of patients, followed by CMML (16%) and MPN (16%) including essential thrombocytopenia (n = 4), primary myelofibrosis (n = 2) and polycythemia (n = 1). MDS/MPNs were present in 8% along with PDGFRα negative hypereosinophilia (8%). AML was associated with true SM in only 5% of cases. Lymphoid AHNMD were rare and constituted only 17% of cases. They were represented mainly by non-Hodgkin's lymphoma (6 patients; 8%) and monoclonal gammapathy of undetermined significance (MGUS) (4 patients; 5%).

Patients' characteristics are detailed in Table 1. Briefly, patients with myeloid AHNMD were older (p = 0.032), had less cutaneous lesions (p = 0.038), splenomegaly (p = 0.003), ascites (p = 0.046) and less bone mineral density (p = 0.045). They had significantly lower hemoglobin level (p = 0.029), higher monocyte counts (p = 0.028), and tryptase (p = 0.046) and LDH (p = 0.020) levels than lymphoid AHNMD. AHNMD was diagnosed concomitantly with SM in 39(67%) patients. For the other patients, the median time from the diagnosis of SM to the diagnosis of the AHNMD was 110 (3–370) months. Of note, in one patient, the diagnosis of polycythemia vera was diagnosed 18 months before the diagnosis of SM was made.

*KIT* mutations were the most frequent alteration, found in 87% of the patients. *TET2* mutation was positive in 12 (27%) cases, only in the myeloid AHNMD group (SM-MDS = 5, SM-CMM = 6; SM-AML = 1). *TET2* positive patients tended to be older (p = 0.08), with more hepatomegaly (p = 0.03), splenomegaly (p = 0.003), ascites p = 0.04), low bone mineral density (p = 0.02). The same patients had lower hemoglobin level (p = 0.01), and had lower platelet counts (p = 0.009) than those negative for *TET2* mutation (table 1). The presence of *TET2* mutation correlated with the presence of *KIT* D816V mutations (p = 0.03) but not with that of *ASXL1* mutations (p = 0.45).

Six (14%) patients (SM-MDS = 3, SM-CMML = 1, SM-AML = 1, SM-PMF = 1) were found positive for *ASXL1* mutations. Patients with lymphoid AHNMD all tested negative for *ASLX1*. Again, *ASXL1* positive patients were not different from *ASXL1* negative patients regarding the main clinical and biological characteristics. *CBL* mutations were found positive in 3 (11%) patients (ET = 1, CMML = 1, MDS/MPN = 1) and *JAK2* Val617Phe mutation in 3 (6%) patients (MDS = 1; ET = 1; PMF = 1).

For the whole group of patients, the median OS time was 85.2 months (58.8–111.7) (Figure S1A). The OS was not statistically different between the lymphoid AHNMD and the myeloid AHNMD groups (median OS: 85.2 *vs* 60months respectively; p = 0.928). However, within the myeloid group, SM-MPN fared better than SM-MDS or SM-AML (median OS: not reached *vs* 79.6 months respectively; p = 0.044,) (data not shown). While median survival time for SM-MPN/SM-hypereosinophilia was not reached, it was of 91.8 (13.2–170.4) months for SM-MDS, of 79.6 (0.1–159.5) months for SM-CMML, of 39.9 (8.5–71.4) months for SM-AML and of 60 (58.8–111.7) months for SM-lymphoproliferative diseases (Figure S1B).

In univariate analysis, the presence of C-findings as defined by the WHO criteria (cytopenia, organomegaly with organ dysfunction, malabsorption, weight loss, hypoalbuminemia) [HR = 3.12; (1.11–8.77); p = 0.030], the AHNMD subtypes (SM-MDS/CMML/AML *vs* SM-MPN/hypereosinophilia) (median OS: 79.66 months *vs* not reached; p = 0.044), Neutropenia [HR = 1.10; (1.02–1.20); p = 0.015], high monocytes level [HR = 1.31; (1.02–1.20); p = 0.015] and the presence of *ASXL1* mutations [HR = 4.91; (1.53–15.71); p = 0.007] had detrimental effects on overall survival.

**Figure 1:**
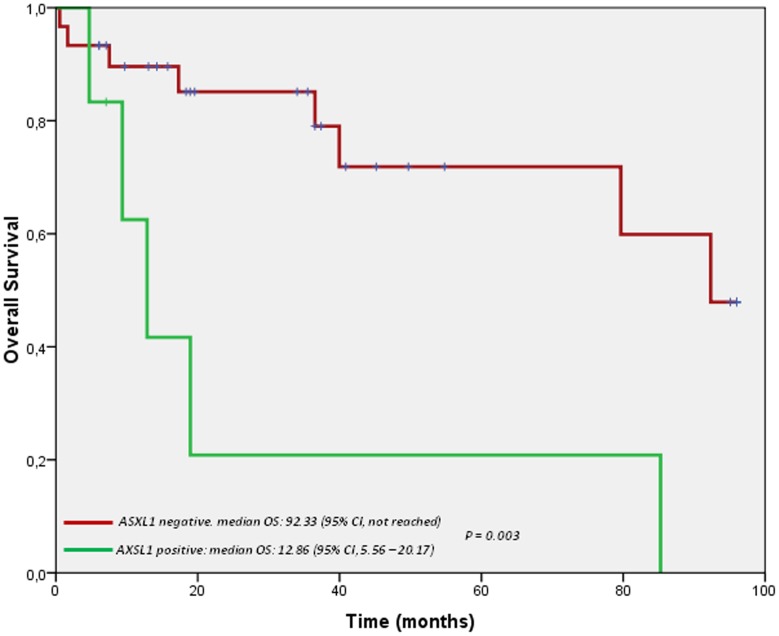
Overall Survival according to *AXSL1* mutations.

In multivariate analysis, as well as, after using penalized Cox regression model [12], only the presence of *ASXL1* mutation remained an independent prognostic factor that negatively affected OS [HR = 5.75; 95%CI (1.09–30.25); p = 0.035] (Figure 1).

This study is, to the best of our knowledge, the second largest study which reports [6], [13]–[15] on SM-AHNMD. *KIT*, *TET2*, *ASXL1*, *JAK2*, and *CBL* were found mutated in 87%, 27%, 14%, 14%, and 11% of the patients respectively.

In this series dedicated to SM-AHNMD subtypes myeloid AHNMD was the most frequent associated disease (82%) where MDS, CMML and MPN represented 29%, 16% and 16% of AHNMD respectively. The median age at the onset of the disease was higher in the myeloid AHNMD than in the lymphoid one, and higher than the median age of mastocytosis patients without AHNMD as already reported [16].

Patients with SM-myeloid AHNMD had more splenomegaly, ascites, low bone density, lower hemoglobin and higher tryptase levels than SM-lymphoid AHNMD patients. However, there was no difference in OS between the 2 groups and this might be explained by the heterogeneity of the subcategories of AHNMD and their prognosis within the myeloid and lymphoid groups.

*KIT* D816V, *JAK2* and *C-CBL* mutations were found in 85%,13% and 11.5% of the patients as already reported [5], [6].

We found *TET2* mutations in 27% of SM-AHNMD patients. This frequency is higher than the incidence encountered in patients with MPN [17] and lower than previously reported in SM-AHNMD in two series of 23 and 8 patients respectively [5], [18].

*TET2* mutated patients tended to be older with more C-findings than patients with wild type *TET2*. However, these findings did not translate into poor prognosis in contrast to the negative prognostic value in cytogenetically normal AML [19], MPNs or mastocytosis patients [20].

Recently, *ASXL1* mutations have been analyzed in only 8 SM patients and found mutated in 2(25%) patients [5]. In our cohort, we found an intermediate frequency of *ASXL1* mutations (14%). They are of prognostic value and shown to adversely affect the OS of patients with SM-AHNMD independently from other clinically utilized prognosticators. *ASXL1* mutations retained also its independent negative impact in the group of patients with SM-MDS/AML (Figure S1C).

As such, mutations in *ASXL1* appear to be novel biomarkers of adverse overall survival in patients with SM-myeloid AHNMD as it has been reported with other myeloid malignancies such as MDS, CMML and subgroups of AML [21]–[23].

### In conclusion

*ASXL1* mutations in SM-AHNMD adversely impact on OS. More studies comparing individual subcategories of SM-AHNMD to their hematological non MCs counterpart without SM are needed.

## Supporting Information

Table S1 **Patient's characteristics.**
(PDF)

Figure S1 **Overall survival (OS) of the whole group (S1A); OS according to the subtype of the AHNMD (S1B); OS of SM-MDS according to ASXL1 mutations (S1C).**
(PDF)
