# Supplementary material for: ASXL1 but Not TET2 Mutations Adversely Impact Overall Survival of Patients Suffering Systemic Mastocytosis with Associated Clonal Hematologic Non-Mast-Cell Diseases
Source: PLoS One. 2014 Jan 21;9(1):e85362. doi: 10.1371/journal.pone.0085362 (PMC3897447; doi:10.1371/journal.pone.0085362)
Supplement: Table S1 — Patient's characteristics. (PDF) [file pone.0085362.s001.pdf]

Supplementary Table 1:

Patient's N°	Sexe	Mastocytosis subtype	AHNMD subtype	KIT mutations' status	ASXL1 mutation	ASXL1 mutation type	TET2 mutation	
#1	Male	ASM-AHNMD	RAEB	D816V	positive	c.1720-1G>C pas d'exon 12	negative	
#2	Male	ISM-AHNMD	MGUS	D816V	WT	na	negative	
#3	female	ISM-AHNMD	ESSENTIAL THROMBOCYTOPENIA	D816V	WT	na	negative	
#4	female	ISM-AHNMD	HYPEREOSINOPHILIA	D816V				
#5	Male	SSM-AHNMD	PRIMARY MYELOFIBROSIS	D816V	WT	na	negative	
#6	Male	ASM-AHNMD	MDS/MPN overlaping syndrome	WT KIT	WT	na	negative	
#7	Male	ISM-AHNMD	Mantle cell lymphoma	D816V				
#8	female	ISM-AHNMD	ESSENTIAL THROMBOCYTOPENIA	D816V				
#9	female	ASM-AHNMD	RAEB	WT KIT	positive	c.2536del p.Ser846Valfs*21	negative	
#10	female	ASM-AHNMD	ESSENTIAL THROMBOCYTOPENIA	D816V	WT	na	negative	
#11	Male	ASM-AHNMD	MDS	D816V	WT	na	positive	
#12	female	SSM-AHNMD	MPN inclassified	D816V	WT	na	negative	
#13	Male	ASM-AHNMD	CMML	D816V	WT	na	positive	
#14	female	ISM-AHNMD	MDS	D816V	WT	na	negative	
#15	Male	ASM-AHNMD	MDS	D816V	WT	na	positive	
#16	female	ISM-AHNMD	POLYCYTHEMIA VERA	D816V			negative	
#17	Male	MCL-AHNMD	CMML	D816V	WT	na	negative	
#18	Male	ISM-AHNMD	HYPEREOSINOPHILIA	D816V				
#19	female	ASM-AHNMD	CMML	D816V	WT	na	positive	
#20	Male	ISM-AHNMD	ESSENTIAL THROMBOCYTOPENIA	D816V	WT	na	negative	
#21	female	ASM-AHNMD	RARS	D816V				
#22	Male		MPN inclassified	D816V	WT	na	negative	
#23	female	ASM-AHNMD	CMML	D816V				
#24	Male	ASM-AHNMD	MDS	D816V	WT	na	positive	
#25	Male	ASM-AHNMD	AML	WT KIT	WT	na	negative	
#26	Male	ISM-AHNMD	HYPEREOSINOPHILIA	D816V				
#27	female	ISM-AHNMD	MGUS	D816V				
#28	Male	ISM-AHNMD	RARS	D816V				
#29	Male	ISM-AHNMD	MDS	D816V				
#30	Male	ASM-AHNMD	RAEB	D816V	WT	na	negative	
#31	Male	ASM-AHNMD	CMML	non D816V	WT	na	positive	
#32	Male	ASM-AHNMD	CMML	D816V	positive	c.1935dup p.Gly646Trpfs*12	negative	
#33	female	ASM-AHNMD	RAEB	D816V	WT	na	negative	

#34	Male	ISM-AHNMD	Waldenström	WT KIT	WT	na	negative
#35	Male	ISM-AHNMD	MDS	WT KIT			
#36	female	ISM-AHNMD	SMALL B CELL LYMPHOMA	D816V	WT	na	negative
#37	Male	SSM-AHNMD	MDS/MPN overlaping syndrome	D816V	WT	na	negative
#38	Male	ISM-AHNMD	MGUS				
#39	Male	ISM-AHNMD	MDS/MPN overlaping syndrome	D816V	WT	na	positive
#40	Male	ISM-AHNMD	Waldenström	WT KIT	WT	na	negative
#41	Male	ISM-AHNMD	LYMPHOMATOIDE PAPULOSIS	D816V	WT	na	negative
#42	Male		RAEB	D816V	WT	na	negative
#43	female	ASM-AHNMD	MDS/MPN overlaping syndrome	D816V	WT	na	positive
#44	female	ISM-AHNMD	SMALL B CELL LYMPHOMA	D816V			
#45	female	ISM-AHNMD	MGUS	D816V	WT	na	negative
#46	female	ISM-AHNMD	HYPEREOSINOPHILIA	D816V			
#47	Male	ASM-AHNMD	MPN inclassified	D816V	WT	na	negative
#48	Male	ASM-AHNMD	MDS/MPN overlaping syndrome	WT KIT			
#49	Male	ISM-AHNMD	HYPEREOSINOPHILIA	D816V			
#50	female	ASM-AHNMD	RAEB	D816V	positive	c.1934dupG p.Gly646Trpfs*12	positive
#51	Male	SSM-AHNMD	CMML	D816V	WT	na	positive
#52	Male	ISM-AHNMD	HAIRY CELL LEUKEMIA	D816V	WT	na	negative
#53	Male	ASM-AHNMD	AML	D816V	WT	na	positive
#54	female	SM-AHNMD	MDS	D816V	WT	na	negative
#55	Male	ASM-AHNMD	AML	D816V	positive	c.1934dupG	negative
#56	female	ASM-AHNMD	MDS	D816V	WT	na	positive
#57	female	ISM-AHNMD	CMML	D816V			
#58	Male	CM-AHNMD	RARS	WT KIT	WT	na	negative
#59	Male	SM-AHNMD	RAEB	D816V	WT	na	negative
#60	Male	ASM-AHNMD	PRIMARY MYELOFIBROSIS	D816V	positive	c.1934dupG	negative
#61	Male	ASM-AHNMD	CMML	D816V	WT	na	negative
#62	female	ASM-AHNMD	CMML	D816V			

Abbreviations: RAEB;refractory anemia with excess of blasts, MGUS; monoclonal gammapathy of undetermined significance, MDS;myelodysplastic syndrome, MPN;myeloproliferative neoplasm, CMML;chronic myelomonocytic leukemia, AML;acute myeloid leukemia, WT;wild type, na;not applicable

Supplementary Figure 1

Suppl Figure 1A: Overall Survival of the whole group

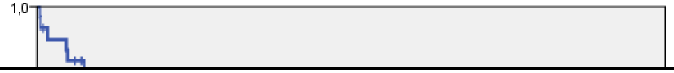

Suppl. Figure 1B: Overall Survival according to the subtype of SM-AHNMD

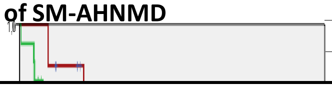

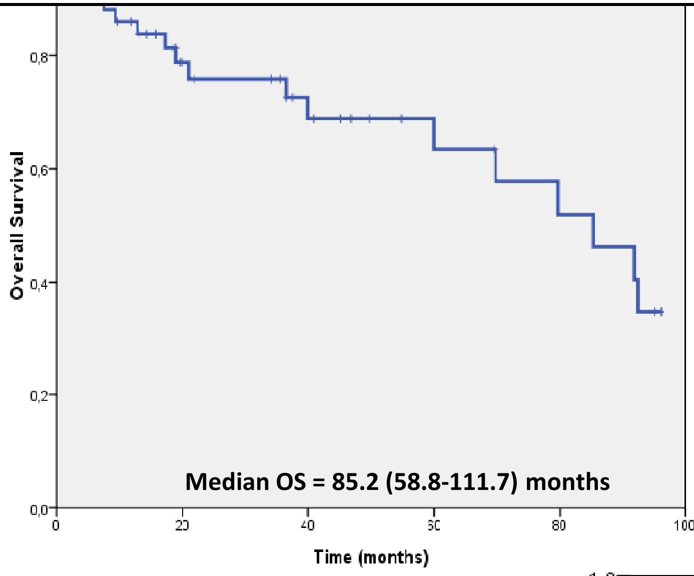

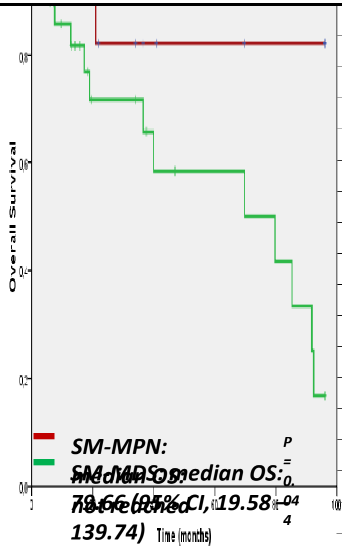

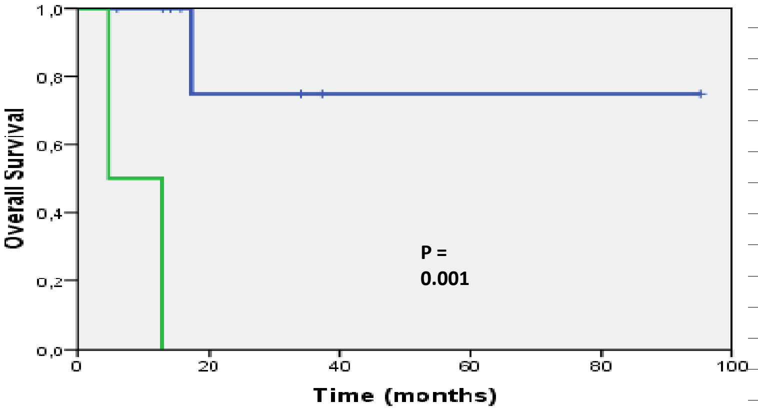

Suppl Figure 1C:
Overall Survival of SM-MDS according to *AXSL1* mutations
