# Supplementary material for: ASXL1 but Not TET2 Mutations Adversely Impact Overall Survival of Patients Suffering Systemic Mastocytosis with Associated Clonal Hematologic Non-Mast-Cell Diseases
Source: PLoS One. 2014 Jan 21;9(1):e85362. doi: 10.1371/journal.pone.0085362 (PMC3897447; doi:10.1371/journal.pone.0085362)
Supplement: Figure S1 — Overall survival (OS) of the whole group (S1A); OS according to the subtype of the AHNMD (S1B); OS of SM-MDS according to ASXL1 mutations (S1C). (PDF) [file pone.0085362.s002.pdf]

Figure S1

Figure S1A: Overall Survival of the whole group

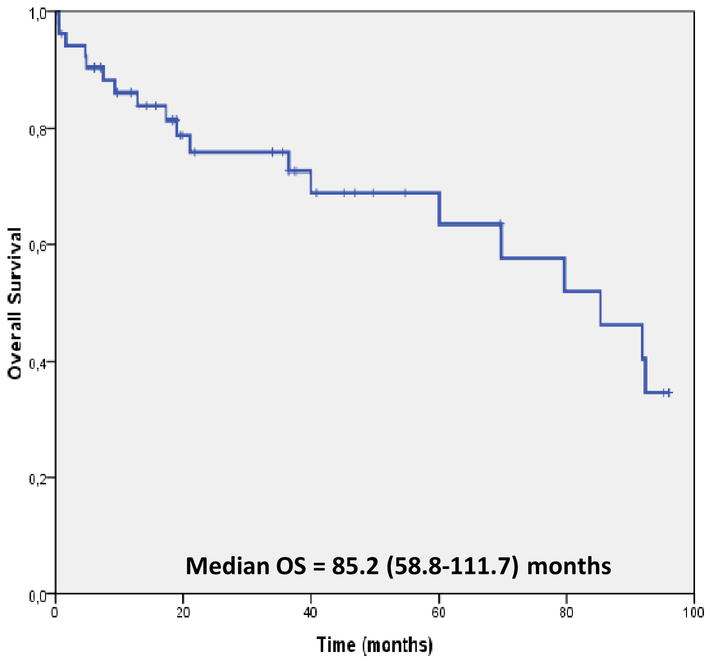

Figure S1B: Overall Survival according to the subtype of SM-AHNMD

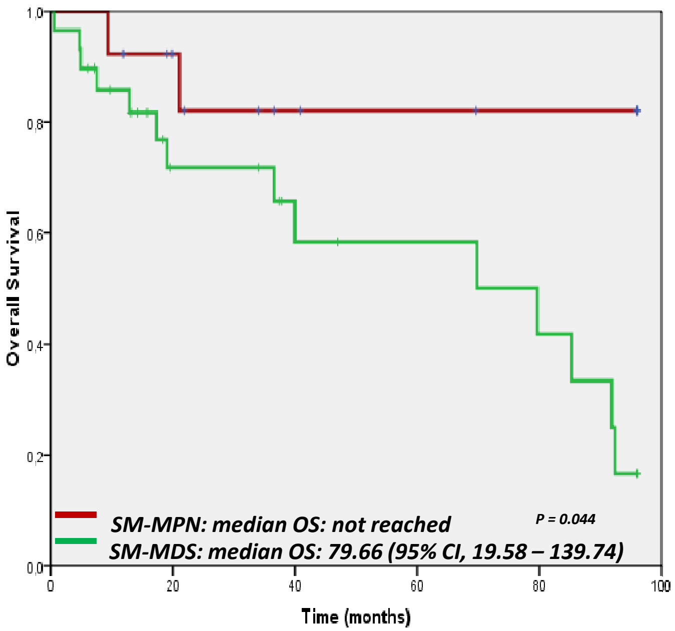

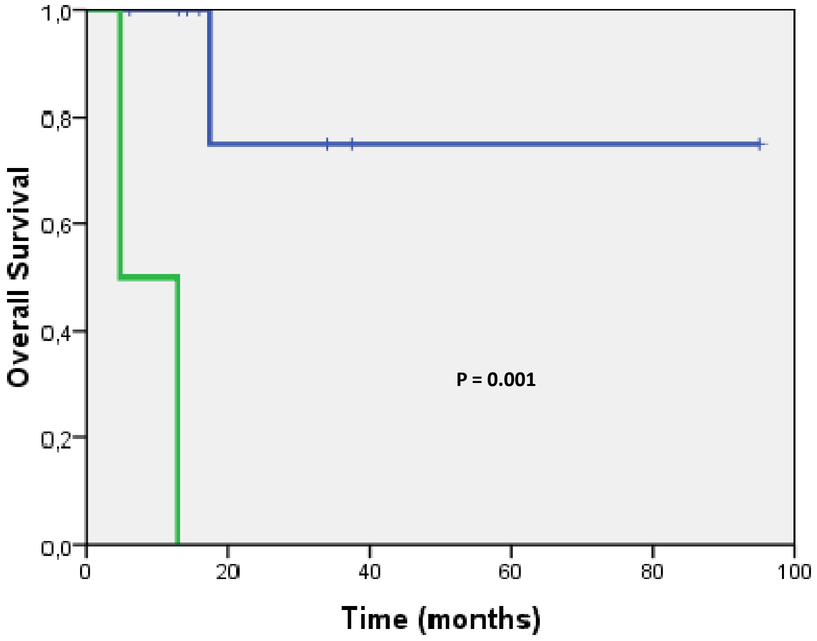

SuppFigure S1C:
Overall Survival of SM-MDS according to *AXSL1* mutations
